# Guided Endodontic Treatment of Calcified Lower Incisors: A Case Report

**DOI:** 10.3390/dj8030074

**Published:** 2020-07-08

**Authors:** Georges Ishak, Marc Habib, Hani Tohme, Shanon Patel, Antonietta Bordone, Cyril Perez, Carla Zogheib

**Affiliations:** 1 Department of Endodontics, Saint Joseph University of Beirut, Beirut 1107-2180, Lebanon; 222georges@gmail.com (G.I.); marc.habib@usj.edu.lb (M.H.); hani.tohme@usj.edu.lb (H.T.); 2 Department of Restorative Dentistry, King’s College, London SE5 9RS, UK; shanonpatel@googlemail.com; 3 Private practice, 13000 Marseille, France; dr.antonietta.bordone@gmail.com; 4 Université de Strasbourg, 6700 Strasbourg, France; dr.perez.cyril@gmail.com

**Keywords:** guided endodontics, calcified teeth, pulp stones, cone beam computed tomography, conservative endodontics, intra-oral scanning, access cavity

## Abstract

A 52-year-old female patient was diagnosed with chronic periapical periodontitis associated with severely calcified lower central incisors. Radiographic examination revealed no visible root canal in the coronal-third of the root. After choosing the guided endodontic treatment, an intraoral scan (Trios, 3shape, Copenhagen, Denmark), in conjunction with a cone beam computed tomography (CBCT) scan, was taken in order to design and fabricate a printed guide. Virtual implant software was used to visualize the surgical access into the sclerosed root canals. After locating the canals, the guide was removed, and the teeth were treated under a rubber dam. The guided approach allows predictable, efficient endodontic treatment of teeth presenting calcified canals, with minimal removal of sound dentine and less risk of root perforations.

## 1. Introduction

Pulp canal calcification, or sclerosis, may be the result of physiological aging of the tooth [1], sequela following dental trauma, surgeries (auto-transplantations), carious lesions, excessive orthodontics, iatrogenic dental treatment [2,3,4,5] or regenerative endodontic procedures [6]. In calcified teeth, the root canal treatment is only indicated in cases of irreversible pulpitis or apical periodontitis [7,8]. In fact, pulpal sclerosis alone is not a reason for endodontic treatment, yet its presence worsens the prognosis of treatment [9,10].

The American Association of Endodontics (AAE) classifies these endodontic treatments as having a high level of difficulty, given the risk of complications or even failures [11]. In such cases, cone beam computed tomography (CBCT) is used to evaluate the endodontic state, the configuration and number of root canals, and most importantly, to estimate a working length [12] and a drilling length specific to each canal [13]. Recently, guided endodontics have been proposed [14]. Thus, its application, including a guide for the drill path [13], a sleeve [15] and a suitable drill [16], could be of potential interest for the management of these complex cases [16,17,18].

## 2. Case Report

**Figure 1:**
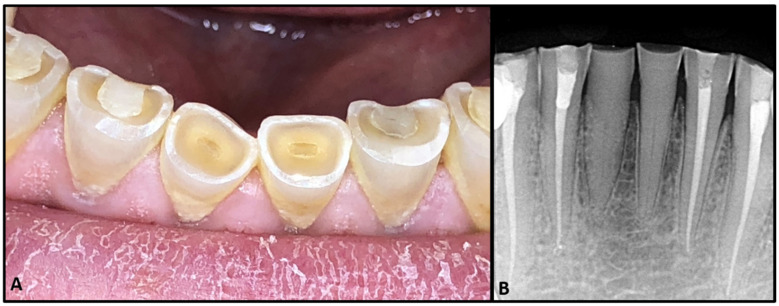
(**A**) Image showing severe tissue loss due to severe bruxism. (**B**) Initial pre-op X-Ray.

**Figure 2:**
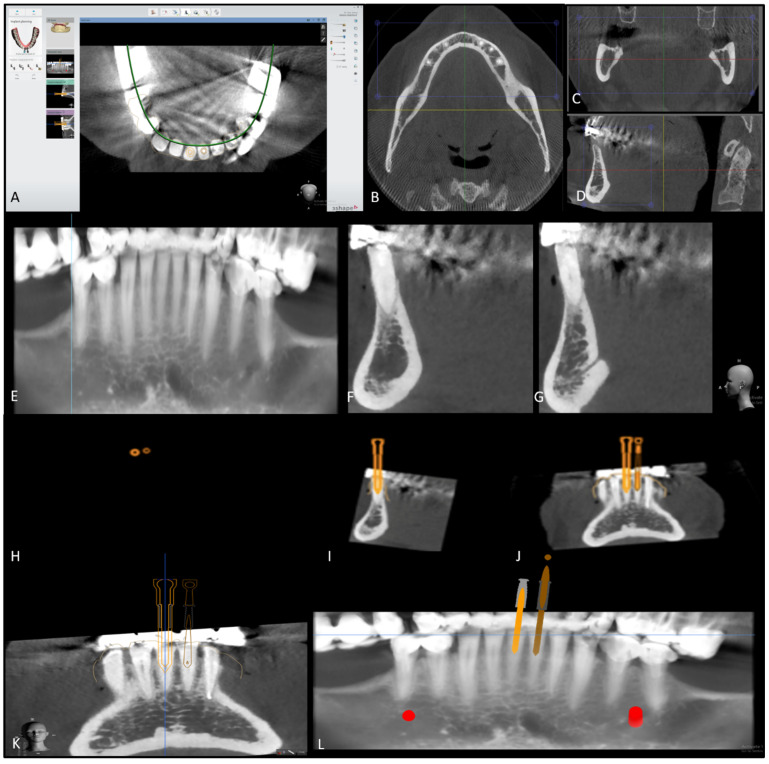
(**A**) Axial view of inferior jaw on Implant studio software. (**B**) Axial view of the mandible. (**C**) Transversal view. (**D**) Sagittal view. (**E**) Panoramic view. (**F**) Sagittal view of tooth 31, showing severe calcification of the canal. (**G**) Sagittal view of tooth 41, showing severe calcification of the canal. (**H**) Axial view of the virtual drill’s preparation. (**I**) Sagittal view of the virtual drill’s preparation and axis. (**J**,**K**) Frontal view of the virtual drill’s preparation and axis. (**L**) Panoramic view of the virtual drill’s preparation and axis.

A 52-year-old patient presented to the dental center of the faculty of dental medicine, Saint Joseph University of Beirut, for management of tooth surface loss. The patient was fit and healthy. Clinically, the teeth were severely worn due to bruxism (Figure 1A), and the patient complained of a dull pain when chewing. Sensibility testing (Endo Ice Refrigerant Spray, Coltene, Ohio, U.S.A.) was negative on teeth 31 and 41; these teeth were tender to percussion. There were no other signs of endodontic or periodontal disease associated with the lower anterior teeth. The radiographic examination (Figure 1B) showed severe calcification of the pulp chamber and root canal, with no apparent radiolucent lesions. After performing a cavity test without anesthesia, both teeth were diagnosed as necrotic, with an early stage of apical periodontitis. Because calcified teeth are categorized as hard cases by the American Association of Endodontics, a high-resolution cone beam computed tomography (CBCT) was first performed in order to evaluate the degree of calcification, the location, and the number of canals. The CBCT scan (VGi evo, NewTom, Verona, Italy, Field of view (FOV) 24 × 19, mA 3.0, kVp 110, Voxel 0.2 mm) revealed visible canal space beyond the coronal and middle third junction, starting at 9 mm for tooth 31 and 4 mm for tooth 41 (Figure 2F,G), measured from the incisal edge of the teeth. Guided endodontic treatment was offered to the patient. The purpose of this report was to evaluate the benefits and limitations of the micro-guided technique.

The patient gave her informed consent before the treatment. This treatment was conducted in accordance with good clinical practice (GCP) established in the declaration of Helsinki, and the council for international organization of medical sciences guidelines (CIOMS). The protocol was approved by the Ethics Committee of “Hotel Dieu de France (HDF)” (Tfemd/2020/28) on March 26, 2020.

**Figure 3:**
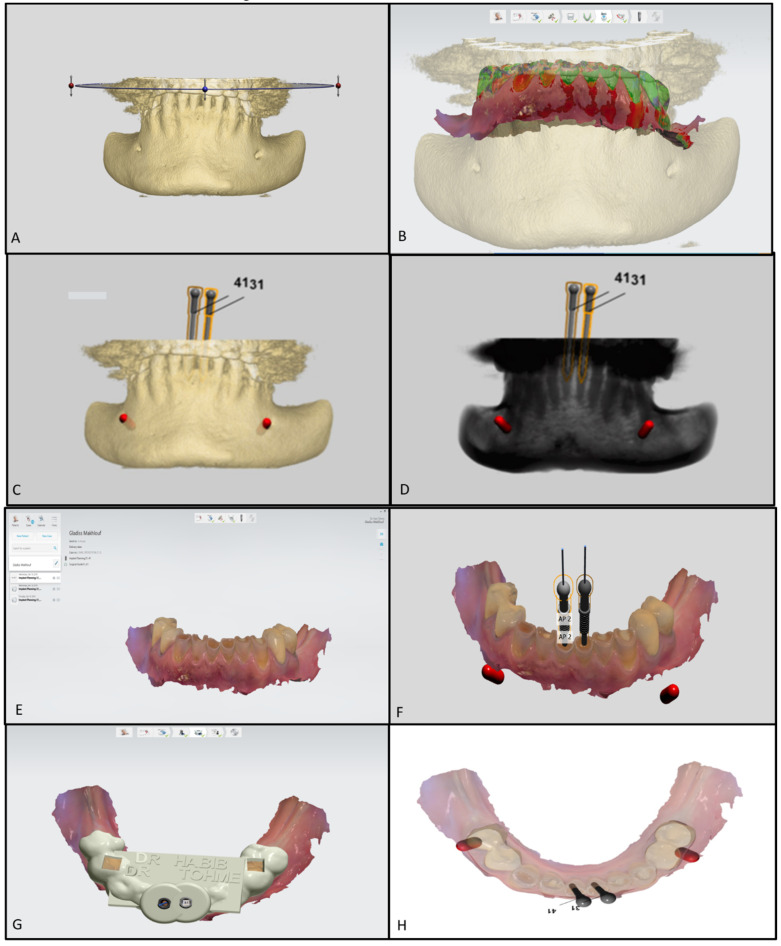
(**A**) 3D reconstruction of the mandibular jaw. (**B**) Superposition of the 3D reconstruction and the digital impression. (**C**,**D**) 3D reconstruction showing the axis of the pins relative to the jaw. (**E**) Digital impression of the inferior arcade. (**F**) Virtual pins’ positions relative to the teeth on the virtual impression. (**G**,**H**) Final model reconstructed.

**Figure 4:**
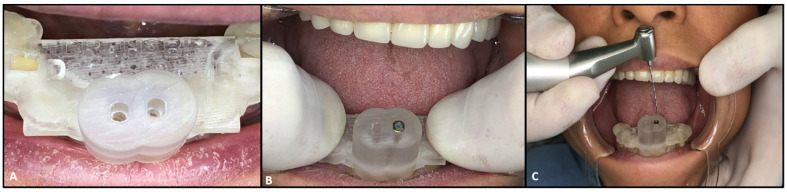
(**A**) Endodontic guide positioning. (**B**) Testing stability and adaptation. (**C**) Drilling through the endodontic guide and sleeve; drill of 0.75 mm diameter (FFDM Pneumonat, Bourges, France).

After the patient’s consent, a 3D intraoral scan was taken of the lower arch (Trios, 3shape, Copenhagen, Denmark) in order to have a 3D virtual model. The intraoral impression was then converted to a 3D stereolithography file (STL file) and imported to Implant studio (3shape, Copenhagen, Denmark), a virtual implant planning software. In addition to the 3D impression, the CBCT images were also uploaded, and both the radiographic and 3D model images were matched on the software. The software was set to design one virtual drill for each tooth. The virtual diameter of the drill was set to 1.5 mm, which is the thinnest virtual drill on the software. However, the actual drill (FFDM Pneumonat, Bourges, France) was 0.75 mm, to fit the dimensions of the central incisors and prevent unnecessary substance loss. The virtual drill was positioned in the axis of the canal, in the center of the tooth, to give a straight access to the canal while maintaining a safety zone of 2 mm apically and 1.5 mm horizontally, hence avoiding any risk of stripping or perforations. After determining the drill’s position, a virtual template of the guide was initiated (Figure 3). A virtual sleeve was also designed in order to maintain the axis during the trepanation of dentin. In contrast with previous guide designs [14], no pins were used to enhance the guide’s stabilization (Figure 4A).

The final model generated was then exported as an STL file and sent to a 3D printer (Asiga Max, Sydney, Australia). The guiding sleeve details of the sleeve (with a 1-mm external diameter, a 0.85-mm internal diameter, and 5-mm length) were separately fabricated to fit the diameter of the guide and drill, and were then incorporated in the guide.

**Figure 5:**
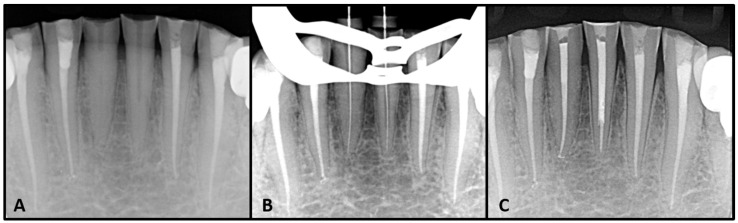
(**A**) Pre-operative X ray after micro-guided drilling. (**B**) Working length determination. (**C**) Post obturation and filling.

The endodontic guide was placed in the patient’s mouth to test its stability and adaptation (Figure 4B). After confirming the guide’s stability, the appliance was removed in order to inject local anesthesia. A small trepanation was made in the center of both teeth with a conical diamond bur (Dentsply Sirona, New York, U.S.A). While this was not mandatory, due to the absence of enamel, the trepanation was made in order to follow the sequence of the treatment and facilitate the drill’s insertion. Subsequently, the drill was used to the estimated calcified lengths of both teeth; 9 mm for tooth 31 and 4 mm for tooth 41 (Figure 4C). A K-type 10 file (M access, Dentsply Sirona, Switzerland) was used to confirm if the canal system could be negotiated. An intra-operative radiograph was then taken to confirm the direction of trepanation and to estimate the residual drilling length needed (Figure 5A). When the root canals became negotiable, a K-type 10 file was used for catheterization. Then, the working length of each tooth was determined electronically, with an apex locator (E-pex, Changzhou Eighteeth Medical Technology, Jiangsu, China), and radiographically: 15 mm for tooth 41 and 15.5 mm for tooth 31 (Figure 5B).

The mechanical preparation of the canals was then carried out with E3 files (E3 Azur Endostar, HT Technology), and a 5.25% sodium hypochlorite solution was used for the chemical preparation. The preparation on tooth 31 shows an apparent ledge on the junction between the middle third and the apical third, approximately 4 mm prior to the radiological apex, due to the drill’s trepanation. An effort to reduce the ledge was carried out by using an instrument with a higher taper, Waveone gold point 25, with 0.07 taper (Dentsply Sirona, New York, U.S.A.). The canals were then obturated with gutta percha and cement (SealiteTM Regular, Acteon Group, Paris, France) with warm vertical compaction (Figure 5C). The access cavities were cleaned and filled with glass ionomer cement, because the patient decided to postpone the prosthetic treatment for financial reasons.

## 3. Discussion

The use of CBCT and computer-aided design and computer-aided manufacturing (CAD/CAM) in dentistry is becoming more and more common in dental practice [12]. CBCT is an undeniable necessity when it comes to hard cases in endodontic treatments. It assists in the diagnosis of periapical lesions and vertical root fractures, in pre-surgery planning, in identifying root canal anatomy [19,20,21], and it gives an estimated degree of canal obliterations and helps identify missed canals [20,22].

This micro-guided endodontic technique improves efficiency [23]. Even when the practitioner does not possess a CBCT and a CAD/CAM machine in-office, this should not be an excuse for not considering the option. CBCT may be of great help in treatment planning because of the information it provides, even if the practitioner chooses to opt for conventional treatment.

This technique is safe, regardless of the amount of calcification, and there is almost no risk of perforation unless there is an error during planning, or a lack of stability in the guide [19]. While the success rate was 80% with the use of ultrasonic tips and microscopes by specialized endodontists [24,25], successful treatments became much more frequent with the micro-guided approach, even when used by general practitioners or unexperienced dentists [26,27]. Therefore, this technique is easy, and done without the need of a dental microscope. However, it does not exclude the risks usually encountered during endodontic treatment (instrument fracture, overextension, ledges, zipping, etc.). The purpose of this technique is to facilitate the access to the canal, and it does not guarantee the success of the treatment.

The difficulty encountered during this treatment was in finding the canal on tooth 31, which required an intraoral radiograph to estimate the position of the canal relative to the drill. The long axis from drilling matched that of the center of the root, and was only 4 mm above the detectable portion of the canal. Since the canal was only detectable in the final third, and the guide’s sleeve inhibited the drill from evolving further apically, the trepanation proceeded without the guide. Nevertheless, the axis was maintained due to the trephined area of the tooth, which served as a guide itself. The canal was found at 12.5 mm with no deviation problems. The use of a thin drill (0.75 mm) is a must, especially in this region, to minimize microcraters and heating of the tooth, which is better than the use of the conventional drills used in past reports [28].

A major inconvenience of this technique is the ledge formation when the calcification extends to more than the junction between the middle third and apical third, as happened with tooth 31. It is important to note here that other reports encountered the same problem [14]. The reason behind this ledge was the necessity of drilling up to the apical third of the root. Since the drill has a diameter of 0.75 mm, or even 1.3 mm in others, it is equivalent to a file 75 with no taper, which is relatively big, and should not be used apically, especially in anterior teeth like inferior incisors. Nevertheless, to overcome such an inconvenience, the drill should not be used beyond the middle of the root canal. However, if the canal was still missing at this point, the use of a more conical-shaped drill would be recommended, like the SSwhite endo guide drills (SSwhite dental, New Jersey, U.S.A.). These drills could be used after the trepanation of half of the canal because the axis of trepanation can now be set by the trephined area of the tooth, which would minimize the risk of axial changes and perforations, compared to the trepanation performed directly with the conical drill. As for tooth 41, no problem was encountered during the negotiation of the canal, as it was found in the middle third of the root and the treatment was performed without any ledges.

Another inconvenience is that this treatment will be more costly for the patient, as they will have to pay for the guide planning and printing. This is why it is important to address the cost with the patient prior to treatment planning. However, the treatment is becoming more and more affordable, as many new software and guide designs are emerging.

## 4. Conclusions

The use of CBCT in calcified teeth is an important aspect of diagnosis and treatment planning. The endodontic guide should be considered during treatment planning for calcified canals, in order to raise the success rate and minimize clinical stress for both the patient and the practitioner. This technique can be used by any dentist who wishes to practice endodontics; however, practitioners should avoid going beyond the middle of the root with the drill, and try conical-shaped drills with a thinner diameter. Further studies are needed to test the efficiency of the use of thin conical drills in the apical third of severely calcified teeth.
